# Construction of gene regulatory networks using biclustering and bayesian networks

**DOI:** 10.1186/1742-4682-8-39

**Published:** 2011-10-22

**Authors:** Fadhl M Alakwaa, Nahed H Solouma, Yasser M Kadah

**Affiliations:** 1 University of Science and Technology, Sana'a, Yemen; 2 Department of Biomedical photonics, Niles, Giza, (12613), Egypt; 3 Department of Biomedical Engineering, Cairo University, Giza, (12613), Egypt

## Abstract

**Background:**

Understanding gene interactions in complex living systems can be seen as the ultimate goal of the systems biology revolution. Hence, to elucidate disease ontology fully and to reduce the cost of drug development, gene regulatory networks (GRNs) have to be constructed. During the last decade, many GRN inference algorithms based on genome-wide data have been developed to unravel the complexity of gene regulation. Time series transcriptomic data measured by genome-wide DNA microarrays are traditionally used for GRN modelling. One of the major problems with microarrays is that a dataset consists of relatively few time points with respect to the large number of genes. Dimensionality is one of the interesting problems in GRN modelling.

**Results:**

In this paper, we develop a biclustering function enrichment analysis toolbox (BicAT-plus) to study the effect of biclustering in reducing data dimensions. The network generated from our system was validated via available interaction databases and was compared with previous methods. The results revealed the performance of our proposed method.

**Conclusions:**

Because of the sparse nature of GRNs, the results of biclustering techniques differ significantly from those of previous methods.

## Background

The major goal of systems biology is to reveal how genes and their products interact to regulate cellular process. To achieve this goal it is necessary to reconstruct gene regulatory networks (GRN), which help us to understand the working mechanisms of the cell in patho-physiological conditions. The structure of a GRN can be described as a wiring diagram that (1) shows direct and indirect influences on the expression of a gene and (2) describes which other genes can be regulated by the translated protein or transcribed RNA product of such a gene [1].

The local topology of a GRN has been used to predict various systems-level phenotypes. For instance, Dyer et al. [2] recently analyzed the intraspecies network of Protein-Protein Interactions (PPIs) among the 1,233 unique human proteins spanned by host-pathogen PPIs. They found that both viral and bacterial pathogens tend to interact with hubs (proteins with many interacting partners) and bottlenecks (proteins that are central to many paths in the network) in the human PPI network.

Within the last few years, a number of sophisticated approaches to the reverse engineering of cellular networks from gene expression data have emerged. These include Boolean networks [3], Bayesian networks [4], association networks [5], linear models [6], and differential equations [7]. The reconstruction of gene networks is in general complicated by the high dimensionality of high-throughput data; i.e. a dataset consists of relatively few time points with respect to a large number of genes. In this study we develop a biclustering function enrichment analysis toolbox (BicAT-plus) to study the effect of biclustering in reducing data dimension.

Clustering algorithms [8-10] have been used to reduce data dimension, on the basis that genes showing similar expression patterns can be assumed to be co-regulated or part of the same regulatory pathway. Unfortunately, this is not always true. Two limitations obstruct the use of clustering algorithms with microarray data. First, all conditions are given equal weights in the computation of gene similarity; in fact, most conditions do not contribute information but instead increase the amount of background noise. Second, each gene is assigned to a single cluster, whereas in fact genes may participate in several functions and should thus be included in several clusters [11].

A new modified clustering approach to uncovering processes that are active over some but not all samples has emerged, which is called biclustering. A bicluster is defined as a subset of genes that exhibit compatible expression patterns over a subset of conditions [12]. During the last ten years, many biclustering algorithms have been proposed (see [13] for a survey), but the important questions are: which algorithm is better? And do some algorithms have advantages over others?

Generally, comparing different biclustering algorithms is not straightforward as they differ in strategy, approach, time complexity, number of parameters and predictive capacity. They are strongly influenced by user-selected parameter values. For these reasons, the quality of biclustering results is also often considered more important than the required computation time. Although some comparative analytical studies have evaluated the traditional clustering algorithms [14-16], no such extensive comparison exists for biclustering even after initial trials have been made [12]. Ultimately, biological merit is the main criterion for evaluation and comparison among the various biclustering methods.

**Figure 1:**
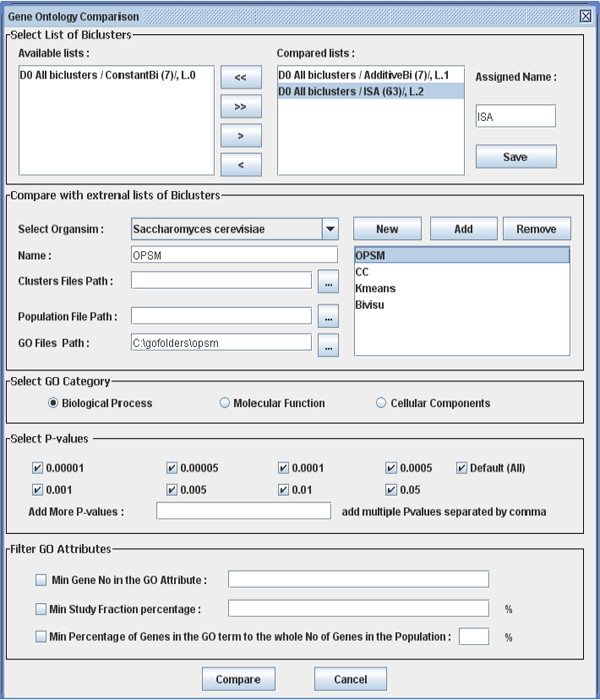
**BicAT-Plus comparison panel**. Algorithms required to be compared could be selected from the biclustering list (left list) to the compared list (right list). External biclustering results for other algorithms could be included in the comparison process. Also the organism model, selectable significance level, and GO category should be selected. Finally, comparison criteria have to be selected on the basis of the user biological metric.

To the best of our knowledge, the biclustering algorithm comparison toolbox has not been made available in the literature. We have developed a comparative tool, BicAT-Plus (Figure 1), that includes comparative biological methodology and is to be used as an extension to the BicAT program [17]. BicAT-Plus and its manual can be downloaded from these two links: http://home.k-space.org/BicAT-plus.zip and http://home.k-space.org/Bicat-plus-manual.pdf. BicAT is a java biclustering toolbox that contains five biclustering and two traditional clustering algorithms.

In this work, one of our goals was to study the value of biclustering algorithms for constructing GRNs.

Bonneau et al. [18] developed a GRN algorithm (The Inferelator) based on an integrated biclustering method (cMonkey) [11]. cMonkey groups genes and conditions into biclusters on the basis of three components: the expression component, the sequence component, and the network component. Not all the biclustering algorithms that are implemented either in BicAT or in our modified version BicAT-Plus required prior information, so we excluded cMonkey from further analysis.

## Methods

### Data Acquisition

Two well-known datasets of yeast microarray gene expression (Gasch et al. [19]; Spellman et al. [20]) were used in this work; they can downloaded from the Stanford Microarray Database (http://smd.stanford.edu/). The Spellman dataset consists of four synchronization experiments (alpha factor arrest, elutriation and arrest of CDC15 and CDC28 temperature-sensitive mutants), which were performed for a total of 73 microarrays during the cell cycle. The Gasch dataset contains 6152 genes and 173 diverse environmental transition conditions such as temperature shock, amino acid starvation, and nitrogen source depletion.

### Preprocessing

Owing to daily *Yeast *chromosomal changes, the experiments of Gasch et al. [19] and Spellman et al. [20] contain genes that no longer exist. We used the SGD Batch Download web tool (http://www.yeastgenome.org/cgi-bin/batchDownload) to remove all the merged, deleted and retired genes from further processing.

Also, microarray measurements may be biased by diverse effects such as efficiency of RNA extraction, reverse transcription, label incorporation, exposure, scanning, spot detection, etc. This necessitates the preprocessing of microarrays prior to data analysis. The datasets used in this work had already been preprocessed for background correction and normalization. Further steps should also be applied for data refinement. In this paper, we applied commonly used preprocessing such as gene filtration and missing value imputation[21,22].

### Data Partitioning

BicAT is an open source tool written in Java swing and containing five biclustering clustering algorithms (OPSM [23], ISA, CC [24], BIMAX [17] and X-motive [25]) as well as two traditional ones (K-means and HCL [26]). The proposed BicAT-Plus adds some features to BicAT. It is flexible and has a well-structured design that can easily be extended to employ more comparative methodologies, helping biologists to extract the best results from each algorithm and interpret them in biologically useful biological ways. The goal of BicAT-plus is to enable researchers and biologists to compare different biclustering methods on the basis of a set of biological merits and to draw conclusions about the biological meaning of the results. BicAT-Plus also helps researchers to compare and evaluate the results of algorithms multiple times according to user-selected parameter values as well as the required biological perspective on various datasets. It adds many features to BicAT, which can be summarized as follows:

• Two more biclustering methods are added: MSBE constant biclustering and MSBE additive biclustering [27]. This enables the package to employ most of the commonly used biclustering algorithms. MSBE is a polynomial time algorithm for finding an optimal bi-cluster with maximum similarity score. We added it because it has the following advantages: (1) no discretization procedure is required, (2) it performs well for overlapping bi-clusters and (3) it works well for additive bi-clusters. When MSBE runs on real data (the Gasch dataset [19]), it outperforms most existing methods in many cases.

• BicAT [17] is extended to perform functional analysis using the three subontologies or categories of Gene Ontology (GO) (biological process, molecular function and cellular component) and visualizing the enriched GO terms for each bicluster in a separate histogram.

• A mean for the evaluation and result display is also added. This feature helps in evaluating the quality of each biclustering algorithm result after the GO functional analysis is applied. It then displays the percentages of enriched biclusters at different significance levels.

• A method for comparing the different biclustering algorithms is also provided. The comparison can be done according to the percentage of the functionally enriched biclusters at the required significance levels, the selected GO category and with certain filtration criteria for the GO terms.

• A further important feature (to be added) is the ability to evaluate and compare the results of external biclustering algorithms. This gives BicAT-Plus the advantage of being a generic tool that does not depend only on the methods employed. For example; it can be used to evaluate the quality of new algorithms introduced to the field and compare them against existing ones.

• The gene ontology enrichment results for each bicluster are visualized using graphical and statistical charts in different modes (2D and 3D). BicAT-Plus provides reasonable methods for comparing the results of different biclustering algorithms by:

• Identifying the percentage of enriched or overrepresented biclusters with one or more GO term per multiple significance level for each algorithm. A bicluster is said to be significantly overrepresented (enriched) with a functional category if the P-value of this functional category is lower than the preset threshold. The results are displayed using a histogram for all the algorithms compared at the different preset significance levels, and the algorithm that gives the highest proportion of enriched biclusters for all significance levels is considered the optimum because it effectively groups the genes sharing similar functions in the same bicluster.

• Identifying the percentage of annotated genes per each enriched bicluster.

• Estimating the predictive power of algorithms to recover interesting patterns. Genes whose transcription is responsive to a variety of stresses have been implicated in a general *Yeast *response to stress (awkward). Other gene expression responses appear to be specific to particular environmental conditions. BicAT-Plus compares biclustering methods on the basis of their capacity to recover known patterns in experimental data sets. For example, Gasch et al. [19] measure changes in transcript levels over time responding to a panel of environmental changes, so it was expected to find biclusters enriched with one of response to stress (GO:0006950), Gene Ontology categories such as response to heat (GO:0009408), response to cold (GO:0009409) and response to glucose starvation(GO:0042149).

### Network Learning

Many reverse engineering approaches to establishing cellular networks from gene expression data have emerged. Bayesian networks (BNs), which were first used by Friedman et al. [4], have been widely used because of their solid basis in statistics. BNs are also able to handle missing data and work with incomplete knowledge about the biological system. There are two important components to representing BNs: the qualitative part, which is called the directed acyclic graph (DAG); and the quantitative part, which is the conditional probability of children given their parents. The popular approach to finding the best DAG is to search the DAG space and find the one with the best score. Because the DAG space is huge, we have to use heuristic searches. K2 algorithm, Greedy Search, Genetic Algorithm and Greedy Hill Climbing are the popular search algorithms. The common objective of these algorithms is to reduce the search space. More about the differences among Bayesian network learning structure algorithms can be found in our previous paper [28].

In this step, we first learn the biclusters produced from different algorithms using the Greedy Hill Climbing search algorithm and BDe Scoring Function implemented in Biolearn [29] at the Department of Biological Sciences, Columbia University.

### Network Generation

After we had obtained all the subnetworks generated from each biclustering algorithm, these subnetworks were integrated by merging new edges and deleting repeated edges to produce the final networks. For examples, for the 219 biclusters generated by the ISA algorithm, learning these biclusters would produce 219 subnetworks. Merging them produced the whole network from the ISA algorithm, which is consisted of 2558 edges.

Finally, we can summarize the procedures in the previous section for generating the final networks as follows:

1. We applied the KNN imputation algorithm [21] to the Spellman dataset in order to substitute the missing data point with the nearest values.

2. All data set genes showing no significant changes were removed.

3. We applied the spectral subtraction denoising algorithm to the dataset [30].

4. Six biclustering algorithms (ISA [31], CC [24], MSBE [27], Bivisu [32], OPSM [23], SAMBA) and one traditional clustering algorithm (k-means) were applied to the Spellman dataset. The total number of biclusters/clusters produced was 683.

5. We ran the Greedy Hill Climbing search algorithm implemented in the Biolearn program [29] to these biclusters and produced 683 subnetworks.

6. These subnetworks were integrated to generate the whole gene network for each biclustering algorithm. When we merged the edges from all the biclustering/clustering algorithms, we produced a big network containing 5440 unique edges. We refer to this network as the *ALL network*.

### Network Analysis and Validation

After the interactions among genes have been inferred, it remains assess whether these relationships exist biologically. It is time and money consuming to validate the full set of predictions experimentally. During the last decade, interaction databases have grown exponentially. More than 230 web-accessible biological pathway and network databases (http://www.pathguide.org) have been reported. These large databases are very promising for assisting GRN inference and validating the inferred networks.

These interaction databases use different identifiers to identify the same gene (GI, SwissProt, internal identifiers, etc.), which requires the resolution of synonymous names/IDs across databases. So, we want to integrate molecular interactions and other types of high-throughput data from different public databases to build biological networks automatically. For this purpose we used BioNetBuilder [33], which is an open-source client-server Cytoscape plug-in that offers a user-friendly interface to create biological networks integrated from several databases. For example, the BioNetBuilder client-server [33] retrieved more than 100,000 interactions for *S. cerevisiae *from different databases as follows: (BIND, 16244); (BioGrid, 99485); (DIP, 17465); (IntAct, 14331); (Interologger, 5395); (KEGG, 5478); (MINT, 11907); the numbers here represent the number of interactions for each corresponding database. Although the network retrieved by BioNetBuilder is still incomplete, we consider it a gold standard network for comparison.

In addition, we have to compare our algorithm's performance via previous methods. In this paper, we compare our algorithm with the Friedman algorithm. Friedman [4] developed a new framework for discovering interactions between genes based on multiple expression measurements that are capable of revealing causal relationships, interactions between genes other than positive correlations, and finer intra-cluster structure. He applied his approach to the dataset of Spellman et al. [20], containing 76 gene expression measurements of the mRNA levels of 6177 *S. cerevisiae *ORFs. (Friedman's network is available from (http://www.cs.huji.ac.il/~nirf/GeneExpression/top800/).

Receiver operator characteristic (ROC) curve and precision recall (PR) curves are commonly used for binary decision problems. We used the DREAM2 [34] evaluation script to compute area and ROC and PR curves. We define some important terms as follows:

• TP: Number of edges present in the gold network and in the predicted network.

• FP: Number of edges not present in the gold network but included in the predicted network.

• FN: Number of edges present in the gold network but not in the predicted network.

• TN: Number of edges not present in the gold network and also not included in the predicted network.

Definitions of TPR, FPR, Recall and Precision can be found in [35].

We also assess the credibility of the network generated by analyzing the network topology using NetworkAnalyzer [36] and finding putative modules using MCODE [37] and BINGO [38].

## Results and Discussion

### Biclustering

We applied BicAT-Plus to the *S. cerevisiae *gene expression data provided by Gasch et al. [19]. The dataset contains 2993 genes and 173 diverse environmental transition conditions such as temperature shock, amino acid starvation, and nitrogen source depletion.

Table 1 shows the biclustering algorithm parameter settings as recommended by the authors in their corresponding publications.

**Table 1:** Parameter settings of biclustering algorithms applied to the Gasch dataset [19].

Algorithm	Parameters	Parameter Description
ISA	tg = 2.0	Genes threshold level
	tc = 2.0	Condition threshold level
	SN = 500	No of seeds

CC	Delta = 0.5	Maximum of accepted score
	Alpha = 1.2	Scaling factor
	M = 100	Number of biclusters to be found

OPSM	l = 100	Number of passed models for each iteration

K-means	M = 100	Number of Biclusters to be found
	IN = 100	Number of Iterations
	RN = 10	Number of replications
	DM = ED	Distance Metric is Euclidean Distance

Bivisu	NT = 0.82	Data Noise threshold
	% NR = 0.33	Minimum % of rows
	NC = 5	Minimum number of columns
	O% = 25%	Maximum overlap allowed

Parameter settings of biclustering algorithms applied to cell cycle gene expression data of *S. cerevisiae *provided by Spellman et al. [20]. For more details about these parameters, see corresponding publication.

Table 2 demonstrates the statistical comparison of the bicluster outputs for each algorithm. They differ in the number of bicluster outputs, the number of genes and conditions within each bicluster, and the ability to recover genes and conditions within its biclusters. CC produces large bicluster size (2259 × 134) because the objective function of this algorithm is to find large biclusters. To that end, it includes an optimization algorithm that maximizes the number of genes within the bicluster and at the same time minimizes the residual, which is the difference between the actual value of an element x_ij _and its expected value as predicted from the corresponding row mean, column mean, and bicluster mean.

**Table 2:** Statistical comparison of bicluster outputs when using the Gasch Dataset [19].

Biclustering Algorithm	No of Biclusters	Bicluster/Cluster Size		GeneCoverage%	ConditionCoverage %
				
		Min	Max		
ISA	9	50 × 35	155 × 37	25	97

CC	69	11 × 5	2259 × 134	100	100

OPSM	2	11 × 15	575 × 6	88.5	32.9

BiVisu	100	27 × 142	99 × 52	55	100

Kmeans	100	20 × 173	50 × 173	100	100

These biclusters were produced when biclustering algorithms were applied with the parameter settings in Table 1 on the Gasch et al. [19] dataset. The size of a bicluster is determined by its number of values, i.e. the product of the numbers of rows and columns; GeneCoverage% and ConditionCoverage% are the percentage of genes, and conditions were recovered for each algorithm respectively.

**Figure 2:**
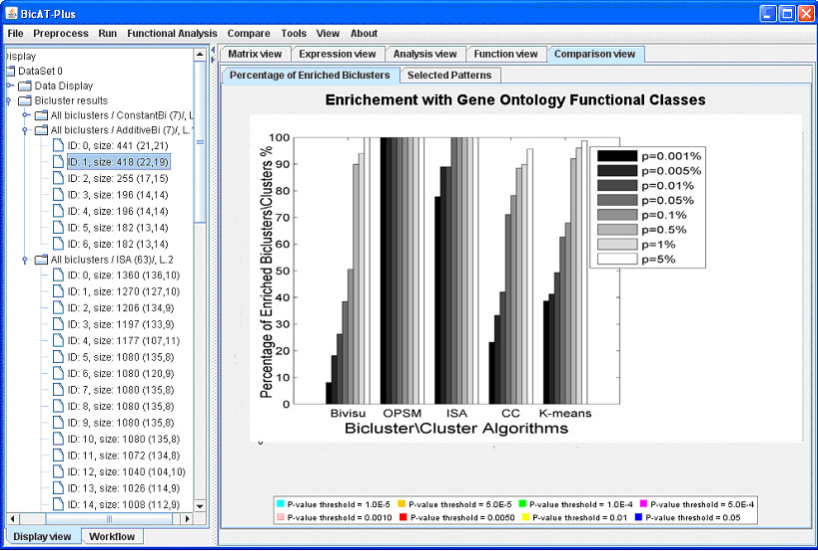
**Percentage of enriched biclusters**. The percentage of enriched biclusters for Biological Process GO annotations (y-axis) is shown against the selected biclustering algorithms (x-axis) at different significance levels. Biclustering algorithms and k-means were applied to the Gasch dataset [19] using the parameter settings in Table 1 with GO annotations of the Biological Process category. A bicluster is said to be significantly overrepresented (enriched) with a functional category if the P-value of this functional category is lower than the preset threshold P-value. The OPSM algorithm gave a high portion of functionally enriched biclusters at all significance levels (from 85% to 100%). Next to OPSM, ISA shows relatively high portions of enriched biclusters.

Comparison of these algorithms using the percentage of enriched biclusters is shown in Figure 2 (histogram). By comparing Figure 2 with Figure 3 in [12,27], we found that the percentages of enriched biclusters for the matched algorithms are almost the same. This validates the results of the proposed comparative tool. Investigating both figures, we observed that the OPSM algorithm gave a high portion of functionally enriched biclusters at all significance levels (from 85% to 100%). Next to OPSM, ISA shows relatively high portions of enriched biclusters.

**Figure 3:**
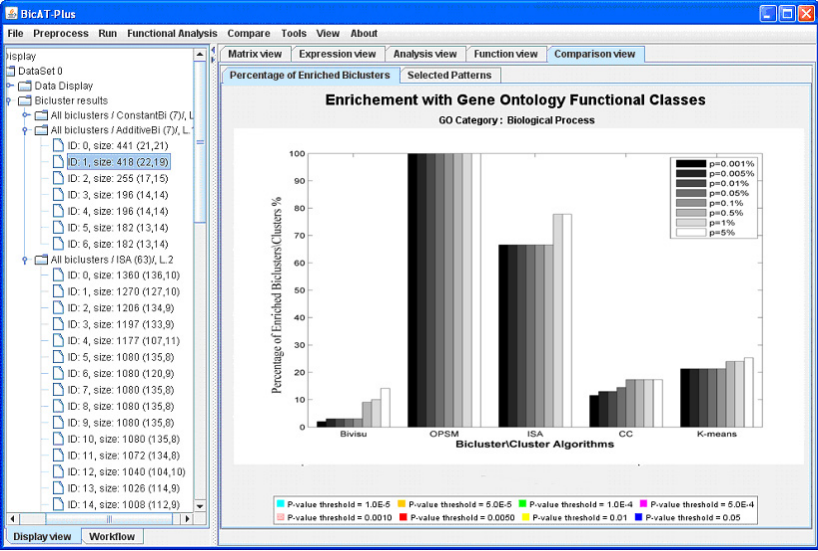
**Percentage of enriched biclusters using restricted criteria**. This Figure is similar to Figure 2 with restriction in the enrichment definition. A bicluster is said to be significantly enriched if the p-value of any of GO category is lower than the preset threshold P-value. The Bivisu and k-means biclusters are strongly affected by this filtration as they contain fewer annotated genes per each category. This filtration criterion helps to identify the most powerful and reliable algorithms that group the maximum numbers of genes sharing the same functions in one bicluster.

In many simulations, we found that most of the enriched biclusters contain few annotated genes. Figure 3 shows the percentage of enriched biclusters in which at least half of their genes are annotated in any GO category. OPSM and ISA have highly enriched biclusters with many annotated genes. In contrast, the Bivisu and k-means biclusters are strongly affected by this filtration as they contain fewer annotated genes in each category. Figure 3 helps to identify the most powerful and reliable algorithms for grouping the maximum numbers of genes sharing the same functions in one bicluster.

Finally, given the ease of comparison allowed by BicAT-Plus, it was straightforward to do further analysis to assess predictive power for recovering interesting patterns; that is, to compare biclustering methods on the basis of which of them recover known patterns in the particular experimental dataset used. Table 3 shows the differences between the bicluster contents based on their predictability to recover the response to stress category. Although OPSM showed a high percentage of enriched biclusters, it had no biclusters with genes matching any of the known GO categories for the Gasch data set. Although there were few ISA biclusters (9) and a low percentage of gene coverage (25%), it showed better performance with one of its biclusters having 11 genes matching response to oxidative stress (GO:0006979). We also see that three methods (k-means, CC and ISA) were able to define biclusters with 4 out of 5 genes in the cellular response to nitrogen starvation functional category, which is very striking. Finally, we observe that several methods appear to be unique in detecting biclusters related to certain function categories. For example, ISA and CC detected two genes belonging to response to cold and cellular response to starvation functions, respectively.

**Table 3:** Comparing biclustering algorithms on the basis of their predictive capacities for recovering selected patterns.

GO Term/number of annotated genes	K-means	CC	ISA	Bivisu	OPSM
GO:0042493 Response to drug (118)	4	5	7	6	0

GO:0006970 Response to osmotic stress (83)	3	5	6	3	0

GO:0006979 Response to oxidative stress (79)	2	7	11	0	0

GO:0046686 Response to cadmium ion (102)	2	3	2	2	0

GO:0043330 Response to exogenous dsRNA (7)	2	3	2	2	0

GO:0046685 Response to arsenic (77)	2	0	2	2	0

GO:0006950 Response to stress (532)	9	11	16	2	0

GO:0009408 Response to heat (24)	3	0	2	2	0

GO:0009409 Response to cold (7)	0	0	2	0	0

GO:0009267 Cellular response to starvation (44)	0	2	0	0	0

GO:0006995 Cellular response to nitrogen starvation (5)	4	4	4	0	0

GO:0042149 Cellular response to glucose starvation (5)	0	2	0	0	0

GO:0009651 Response to salt stress (15)	2	7	0	0	0

GO:0042542 Response to hydrogen peroxide (5)	0	0	0	2	0

GO:0006974 Response to DNA damage stimulus (240)	0	22	0	3	0

GO:0000304 Response to singlet oxygen (4)	2	0	0	0	0

We tested the predictive capacities of different biclustering algorithms for recovering the gene ontology category within response to stress (GO:0006950). Rows represent the known gene ontology function categories under the response to stress category and the columns are tge different biclustering methods, with the highest relevant performance cluster result as the entry for a given functional category and clustering method. Several interesting observations can be made. First, although OPSM showed a high percentage of enriched biclusters, it had no biclusters with genes matching any of the known GO categories for the Gasch data set. Second, despite the low number of biclusters (9) and low percentage of gene coverage (25\%), ISA showed better performance, with one of its biclusters having 11 genes matching response to oxidative stress (GO:0006979). Finally, we observe that several methods appear to be unique in detecting biclusters related to certain function categories. For example, ISA and CC detected two genes belonging to the response to cold and cellular response to starvation functions, respectively.

The comparison methodology used in this study indicates that the present methods show no clear winner, and in fact it seems that all methods should somehow be integrated together to capture the information in the data (i.e. biclustering algorithms differ in strategy, approach, time complexity, number of parameters and predictive capacity, so we expect that each algorithm can recover what other algorithms cannot. So on inspection of Table 3, we recommend biologists to run all biclustering algorithms on their data set and select the enriched results.)

As Friedman used the Spellman [20] cell cycle dataset, we applied BicAT-Plus to this dataset. We used the parameter settings shown in Table 4 and produced the biclusters shown in Table 5. One remarkable observation is that the gene coverage percentage of the ISA algorithm differs from the Spellman dataset (91%) (see Table 5) and the Gasch dataset (25%) (see Table 2). This confirms that each dataset has its unique signature, so integrating more than one dataset enables biological knowledge to be extracted that could not be extracted from a single dataset.

**Table 4:** Parameter settings of biclustering algorithms applied to the Spellman dataset [20].

Algorithm	Parameters	Parameter Description
ISA	tg = 2.0	Gene threshold level
	tc = 2.0	Condition threshold level
	SN = 500	No of seeds

CC	Delta = 0.5	Maximum accepted score
	Alpha = 1.2	Scaling factor
	M = 100	Number of biclusters to be found

OPSM	l = 100	Number of passed models for each iteration

K-means	M = 100	Number of biclusters to be found
	IN = 100	Number of Iterations
	RN = 10	Number of replications
	DM = ED	Distance Metric is Euclidean Distance

Bivisu	NT = 0.5819	Data noise threshold
	% NR = 1.57	Minimum% of rows
	NC = 5	Minimum number of columns
	O% = 25%	Maximum overlap allowed

MSBE	alpha = 0.4	Similarity threshold
	beta = 0.5	Bonus similarity threshold
	gamma = 1.2	Threshold of the average similarity score

SAMBA	MHS = 100	Maximal memory allocated for hashing
	KHS1 = 4	stage
	PC = 100	Maximal kernel size in the hashing stage
	KHS2 = 4	Minimal number of responding probes per condition
	O% = 25%	Minimal kernel size in the hashing stage Maximum overlap between two biclusters

Parameter settings of biclustering algorithms applied to cell cycle gene expression data of *S. cerevisiae *provided by Spellman et al. [20]. For more details about these parameters, see the corresponding publication.

**Table 5:** Statistical comparison of bicluster outputs using the Spellman dataset [20].

Biclustering Algorithm	No of Biclusters	GeneCoverage%	ConditionCoverage%
Kmeans	100	100	100

ISA	219	91	100

CC	69	100	100

OPSM	12	84	57

BiVisu	100	62	100

These biclusters were produced when biclustering algorithms were applied with the parameter settings in Table 4 to the Spellman et al. [20] data set. GeneCoverage% and ConditionCoverage\% are the percentages of genes and conditions recovered per each algorithm respectively.

### Network Validation

**Figure 4:**
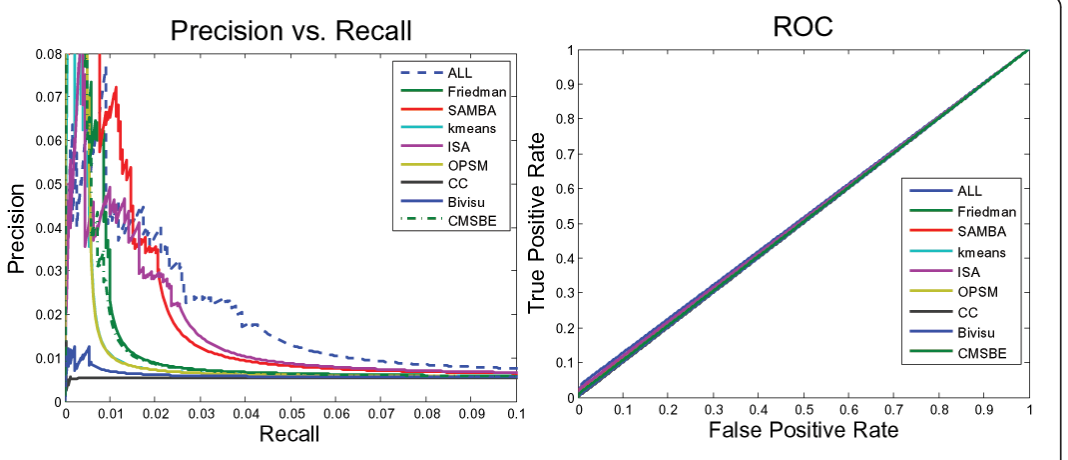
**ROC and PR curves of different biclustering networks that have learned using Bayesian networks **[29]. A performance comparison of networks generated from learning corresponding biclustering algorithms using the Bayesian networks method via the Friedman network [4] and the gold network retrieved by BioNetBuilder [33]. This figure shows that most of these networks contained few true positive edges. Neither the networks generated from different bicluster algorithms nor those generated from all biclustering networks (dashed line) perform well. ALL: This network is produced by integrating edges from all biclustering networks; Friedman Network [4]; SAMBA: This network is generated by integrating SAMBA [43] subnetworks; Kmeans: This network is generated by integrating k-means subnetworks; ISA: This network is generated by integrating ISA [31] subnetworks; OPSM: This network is generated by integrating OPSM [23] subnetworks; CC: This network is generated by integrating CC [24] subnetworks; Bivisu: This network is generated by integrating Bivisu [32] subnetworks; CMSBE: This network is generated by integrating MSBE [27] subnetworks.

Figure 4 and Table 6 show the performance of the biclustering networks via the gold network retrieved by BioNetBuilder [33] and the Friedman network [4]. Inspecting Figure 4 and Table 7, we find that neither the networks generated from different bicluster algorithms nor the *ALL network *perform well. There are two important considerations when interpreting the results of this comparison. First, the interactions documented are either physical or genetic. This implies that they may not be direct interactions. The precision may be lower than the actual precision since links may be missing in the interactome databases; and the recall may be lower than the actual recall in part because some of the links reported in the interactome databases may be indirect [39]. Second, some presently unsupported edges in the constructed network may find experimental evidence in the future. Therefore, these unsupported edges are not necessarily false [40].

**Table 6:** Number of network edges generated from different biclustering algorithms and Friedman network.

Bicluster Network	Number of Edges
K-means network	380

ISA network	2558

OPSM network CC network	220 590

Bivisu network	1515

MSBE network	735

SAMBA network	1611

Total Number of Edges(ALL Network)	5440

Friedman Network	947

For example, applying the ISA algorithm to the Spellman dataset produced 219 biclusters (Table 5); learning these biclusters produced 219 subnetworks. Integrating them produced the whole ISA network, which contains 2558 edges.

**Table 7:** Performance comparison of the biclustering networks.

Methods	EdgeCount	TP	FP	TN	FN	AUROC	AUPR
Gold	2194	2194	0	400396	0	1	1

ALL	5440	94	5346	395050	2100	0.5148	0.0073

SAMBA	1611	46	1565	398831	2148	0.5085	0.0072

ISA	2558	56	2502	397894	2138	0.5097	0.0067

OPSM	220	12	208	400188	2182	0.5025	0.0067

Friedman	947	22	925	399471	2172	0.5039	0.0065

CMSBE	735	20	715	399681	2174	0.5037	0.0063

K-means	380	13	367	400029	2181	0.5025	0.0061

Bivisu	1515	13	1502	398894	2181	0.5011	0.0055

CC	590	3	587	399809	2191	0.5000	0.0054

Performances of biclustering networks are compared with the Friedman network and gold network. EdgeCount: the number of network edges; TP: number of true positive edges; TN: number of true negative edges; FP: number of false negative edges; AUROC: area under ROC curve; AUPR: area under precision recall curve.

For the above reasons, the False Positive (FP) edges could be considered True Positive (TP) if supporting evidence were found in the interaction databases (gold network). For example, if the inference network includes an edge between gene1 and gene3, which does not exist in the gold network, and if these two genes were connected indirectly via another intermediate gene such as gene2, we can now consider the edge between gene1 and gene3 to be a true positive edge. To be entirely consistent we change TN edge into a FN every time there is an interaction via an intermediate gene.

**Figure 5:**
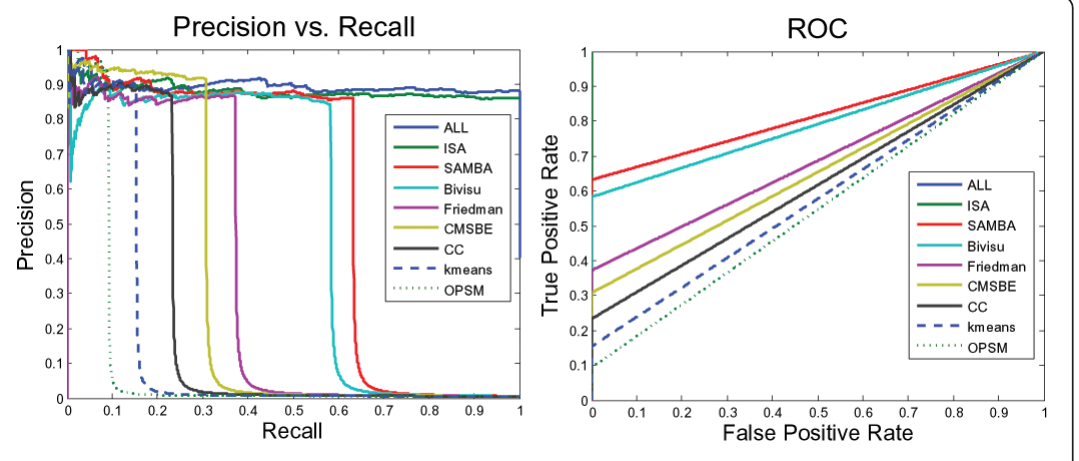
**ROC and PR curves of biclustering networks using modified evaluation methodology**. The poor result of the previous figure (Figure 4) should be considered with regard to two important issues. First, some of the links reported in the interactome databases may be indirect rather than the direct. Second, the available interactome databases are still incomplete, so the false positive edges that are unsupported are not necessarily false and may find experimental evidence in the future. The False Positive (FP) edges could be considered True Positive (TP) if they have evidence in the literature (gold network). For example, if the inference network includes an edge between gene1 and gene3, which does not exist in the gold network, and if these two genes connect indirectly via another intermediate gene such as gene2, we can now consider the edge between gene1 and gene3 as a true positive edge. To be entirely consistent we change a TN edge into FN every time there is an interaction via an intermediate gene.

Table 8 and Figure 5 show the improvement in performance of the networks after taking the above evaluation modification into consideration. Furthermore, they show how most of the false positive edges in these networks have evidence in the gold network (the seventh column in Table 8).

**Table 8:** Performance comparison of the biclustering network using new evaluation criteria.

Methods	EdgeC ount	TP	FP	TN	FN	FP to TP	TN to FN	AURO C	AUPR
Gold	2194	2194	0	400396	0	0	0	1	1

ALL	5440	94	5346	395050	2100	4623	2150	0.7530	0.4614

SAMBA	1611	46	1565	398831	2148	1340	2501	0.6958	0.3644

ISA	2558	56	2502	397894	2138	2141	1151	0.8451	0.6306

OPSM	220	12	208	400188	2182	190	2453	0.5423	0.089

Friedman	947	22	925	399471	2172	794	2700	0.6364	0.2491

CMSBE	735	20	715	399681	2174	653	2750	0.6181	0.2333

K-means	380	13	367	400029	2181	323	3100	0.5667	0.1307

Bivisu	1515	13	1502	398894	2181	1265	2610	0.6845	0.3326

CC	590	3	587	399809	2191	507	2800	0.5943	0.1788

The performances of the various biclustering algorithms improved when false positive edges could be considered true positive edges on the basis of strong evidence in the gold network. Column 7 shows the number of false positive edges in each algorithm that could be considered true positive edges (i.e. have evidence in the gold standard network). Column 8 shows the number of true negative edges that are considered as false negative.

It should be mentioned that, as we expected, the sparse nature of the GNR makes biclustering techniques (ISA, SAMBA, Bivisu) outperform the Friedman network. This promotes the use of biclustering algorithms to overcome the dimensionality problem in GRN inference.

As the success of biclustering algorithms in grouping functionally related genes (i.e. producing highly enriched biclusters), the corresponding learned subnetworks contain many true positive edges. This explains the performance difference in Table 8. So the challenge to produce a real network is reflected in finding enriched biclusters. Figures 2 and 3 and table 3 explain the high and low performance of algorithms ISA and OPSM, respectively. As ISA produces highly enriched biclusters (Figures 2 and 3) and is able to recover the selected pattern (Table 3), it produced a more realistic network; the opposite was the case for the OPSM algorithm. On the other hand, the ISA network even outperforms the SAMBA network: SAMBA produces fewer biclusters than ISA and recovers a lower percentage (see Table 5).

**Figure 6:**
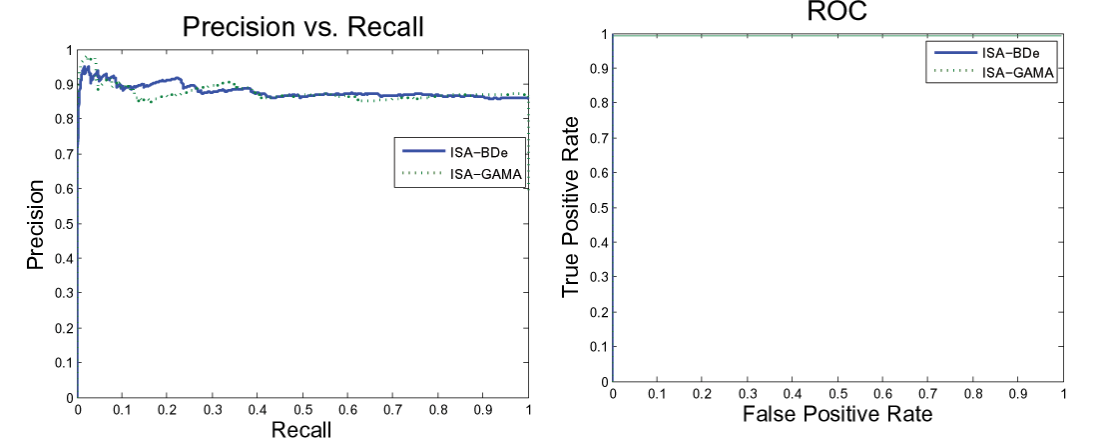
**ISA network performance using different scoring function**. This figure suggests that the ISA network performs equally using the NormalGamma (dotted line) and BDe (solid line) scoring functions.

**Figure 7:**
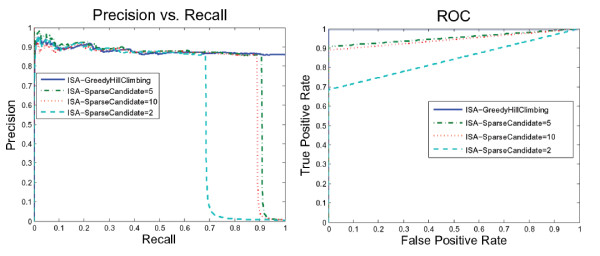
**ISA network performance using different search algorithms**. Network performance using Greedy Hill Climbing outperformed the SparseCandidate learning algorithm. For the SparseCandidate algorithm, decreasing or increasing the size of the candidate sets beyond five worsens the network performance.

We also tried more than scoring functions. Figure 6 suggests that the ISA network performs equally using NormalGamma and the BDe scoring function. On the other hand, Figure 7 demonstrates that the ISA network using GreedyHillClimbing outperformed the SparseCandidate algorithm with a different size of candidate sets. Furthermore, decreasing or increasing the size of the candidate sets beyond five affects the network performance negatively.

To examine whether the performance on the datasets is typical of all network reconstruction methods and is not particular to Bayesian networks with biclustering, we ran another construction algorithm (linear regression) and compared the resultant networks with those generated from the Bayesian networks method. We used the LASSO algorithm, which is implemented in Faisal et al. [41] at the Helsinki Institute for Information Technology (http://users.ics.tkk.fi/faisal/Softwares/LassoRegression.tar.gz).

**Figure 8:**
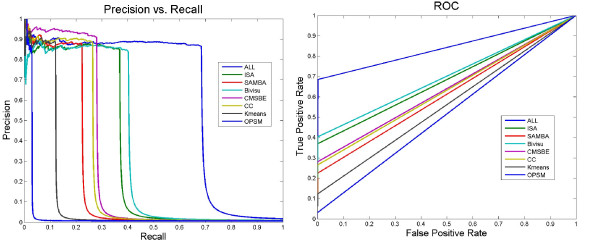
**ROC and PR curves of different biclustering networks that have learned using linear regression method **[41]. A performance comparison of networks generated from learning corresponding biclustering algorithms using linear regression via the gold network retrieved by BioNetBuilder [33]. Comparing Figure 8 with the Bayesian results in Figure 5, we find that the performance of the CMSBE [27] network does not change significantly; the performances of the ALL (this network is produced by integrating edges from all biclustering networks), OPSM [23] and Bivisu [32] networks are greater using the LASSO method than the Bayesian networks method; and the performances of the ISA [31], SAMBA [43] and K-means, networks are lower using the LASSO method than with the Bayesian networks method. We may conclude from Figures 5 and 8 that while different network reconstruction algorithms will lead to differences in the absolute performance, different biclustering schemes consistently have similar relative performances, irrespective of the network reconstruction algorithm used.

We used the cross-validation method to determine the best optimum lambda. Figure 8 shows network performances using linear regression. Comparing Figure 8 with the Bayesian results in Figure 5, we find the following:

• The performance of the CMSBE network does not change significantly.

• The performances of the ALL, OPSM and Bivisu, networks are greater using the LASSO method than with the Bayesian networks method.

• The performances of the ISA, SAMBA and K-means, networks are lower using the LASSO method than with the Bayesian networks method.

We could conclude from Figures 5 and 8 that while different network reconstruction algorithms will lead to differences in absolute performance, different biclustering schemes consistently have similar relative performances, irrespective of the network reconstruction algorithm used. 

Furthermore, analyzing network topology increases the credibility of the predicted network. We therefore analyzed the ISA network and the gold network using NETWORKANALYZER [36]. Table 9 shows that these three important parameters are the same in the two networks, indicating the high performance of the ISA network.

**Table 9:** Analyzing the topologies of the ISA and gold networks using NetworkAnalyzer[36].

Parameters	Gold Standard Network	ISA Network
Network Diameter	8	9

Network Density	0.011	0.012

Avg. no of neighbors	6.91	6.933

NetworkAnalyzer performed topological analysis on the ISA and gold networks. Topological network parameters have biological meaning in regard to the propagation of information among genes. The closeness in the parameters of the ISA and gold networks indicates the credibility of the ISA network.

**Figure 9:**
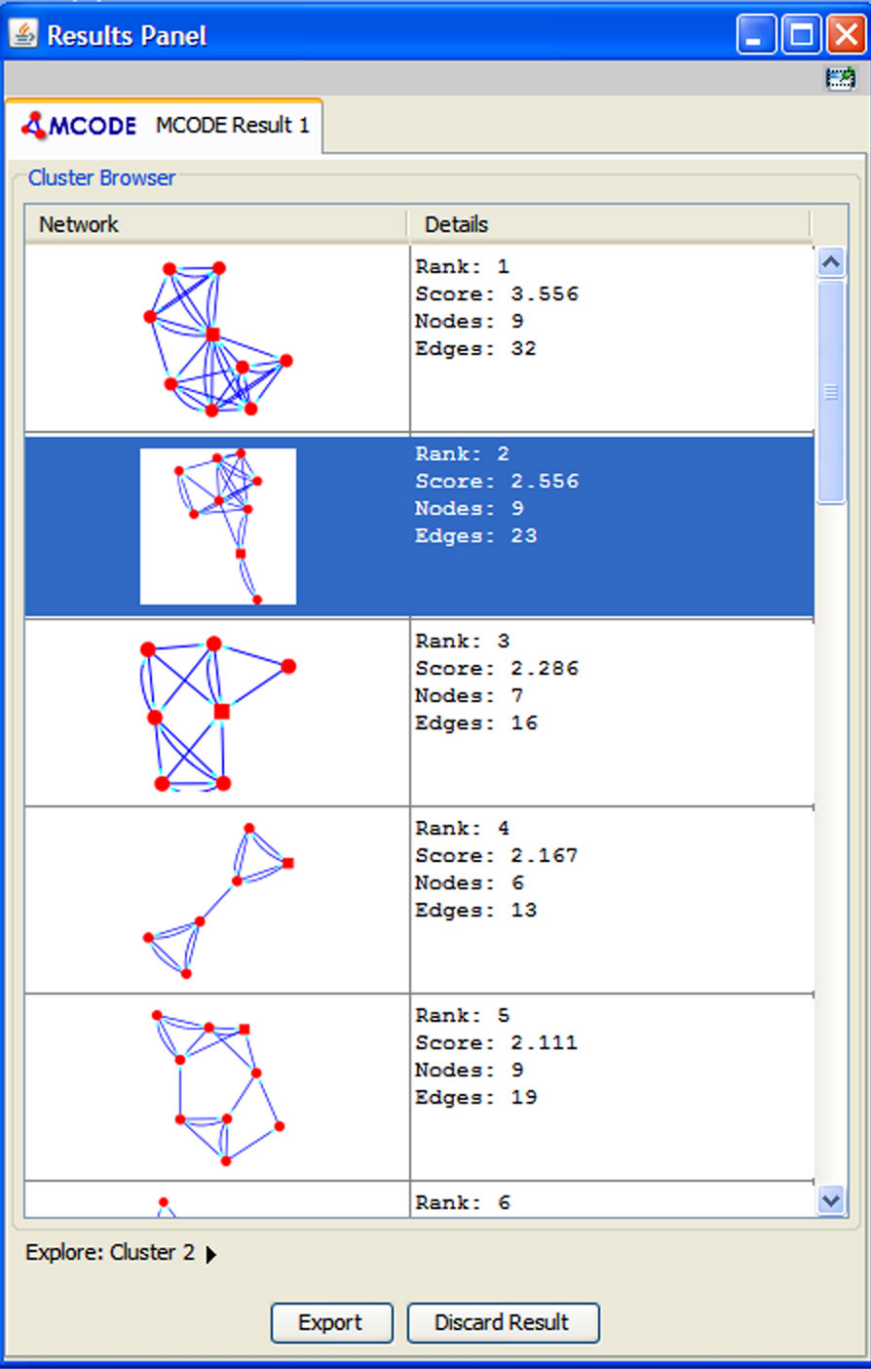
**Detecting modules of ISA network using MCODE **[37]. We used the MCODE Cytoscape plug-in [37] to detect densely connected regions in the ISA network, and this retrieved 39 modules (five are shown). MCODE finds putative complexes through network connectivity. This figure shows the highly scoring modules with the number of nodes and edges and the topology of each module discovered. A significant number of modules have high scores and few nodes and edges.

**Figure 10:**
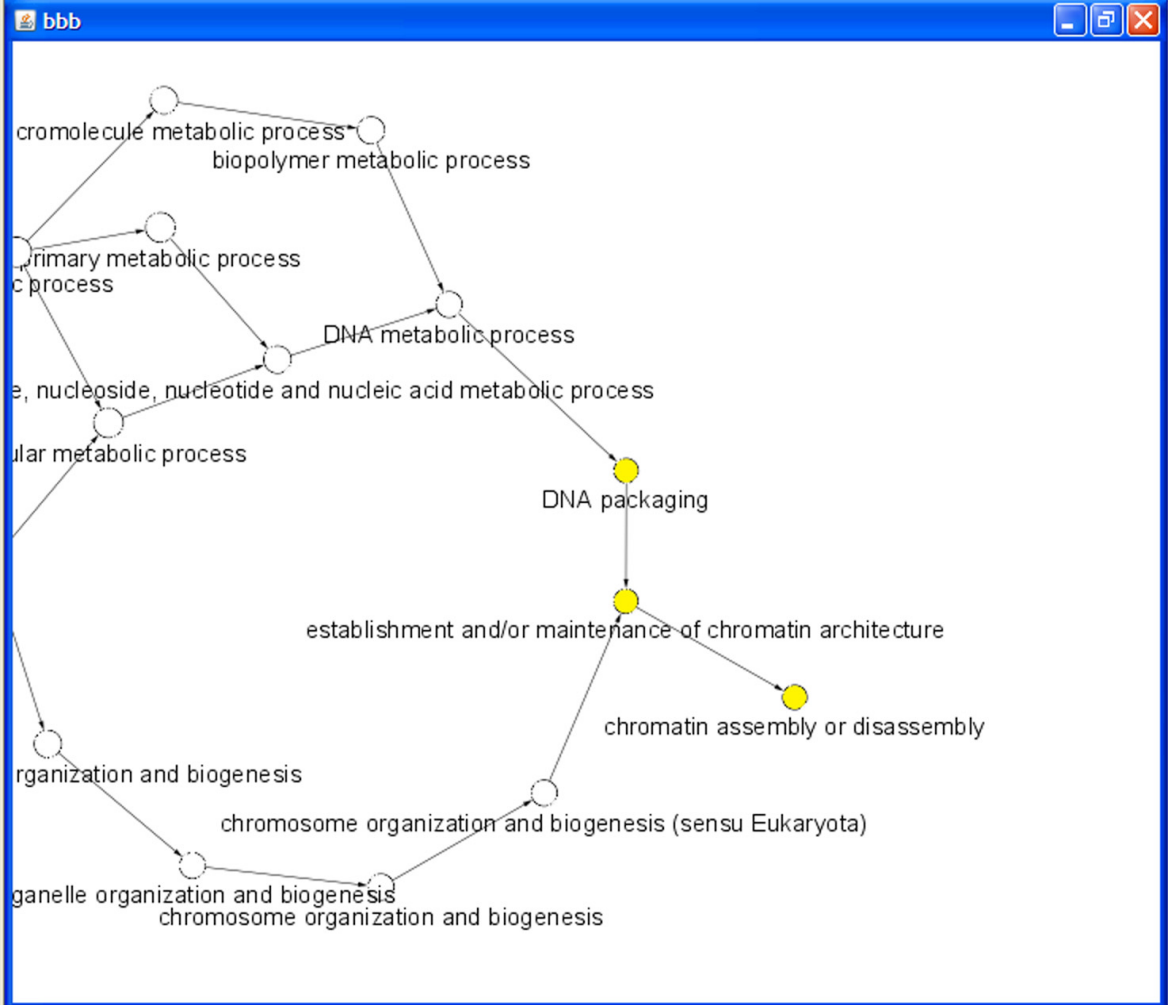
**Functional enrichment of highly scored ISA network module**. To validate the significance of the modules recovered by MCODE [37], the highly scored module (with Rank 1 in Figure 8) was validated using the functional enrichment analysis by the BINGO program [38]. The figure indicates that module genes share three related biological process: Chromatin assembly or disassembly, DNA Packaging and Establishment, and/or Maintenance of Chromatin Architecture.Uncolored nodes are not overrepresented, but they are the parents of overrepresented categories further down. Yellow nodes represent GO categories that are overrepresented at the significance level.

Finally, one of the best methods for validating a network is to assess its accumulated information using the information published in the biological literature. Clustering algorithms have been used to identify molecular complexes or modules in a large protein interaction network through network connectivity [37]. A network module is a group of nodes in the network that work together to execute some common function. We used the MCODE Cytoscape plug-in [37] to detect densely connected regions in the ISA network, which retrieved 39 modules. Figure 9 shows the highly scored modules with the number of nodes and edges and the topology of each module discovered. To validate the significance of the recovered modules, their nodes are a portion of a complex, so there should be some process in which they all operate. Thus, if we explore Gene Ontology (GO) term enrichment using functional enrichment tools such as BINGO [38], we should see some biological process with significant enrichment for these nodes [42]. Figure 10 demonstrates the functional enrichment of a highly scored module using BINGO [38], which indicated that the module genes share three related biological process: Chromatin assembly or disassembly, DNA Packaging and Establishment and/or Maintenance of Chromatin Architecture.

## Conclusions

The ongoing development of high-throughput technologies such as microarray prompts researchers to study the complexity of gene regulatory networks (GRNs) in cells. GRN inference algorithms have significant impact on drug development and on understanding of disease ontology. Many GRN inference algorithms based on genome-wide data have been developed to unravel the complexity of gene regulation. Transcriptomic data measured by genome-wide DNA microarrays are traditionally used for GRN modelling. This is because RNA molecules are more easily accessible than proteins and metabolites. One of the major problems with time series microarrays is that a dataset consists of relatively few time points with respect to a large number of genes. Reducing the data dimensions is one of the interesting problems in GRN modelling. The most common and important design rule for modelling gene networks is that their topology should be sparse. This means that each gene is regulated by only a few other genes. In this work, a new gene regulatory network (GRN) construction system from a large microarray dataset and prior biological information was proposed. As we expected, because GRNs are sparse, biclustering techniques show significant results compared to the Friedman network [4]. In this paper, we show the impact of using biclustering algorithms in GRN construction. Sophisticated filtration procedures such as data filtration, missing value imputation, normalization and discretization were used to reduce the number of expression profiles to some subset that contains the most significant genes.

Also, the biclustering comparison toolbox (BicAT-Plus) implemented in this paper confirms that the bicluster and cluster algorithms can be considered as an integrated module; there is no single algorithm that can recover all the interesting patterns. What algorithm A recovers in certain data sets, Algorithm B might fail to recover, and vice versa. We can identify the highly enriched biclusters in all the algorithms compared, integrating them to solve the dimensionality problem of GRN construction.

Moreover, the study in this paper confirms the ability of Bayesian Networks (BNs) structure algorithms to recover gene network structures accurately. BNs allow us to deal with the noise inherent in experimental measurements and to model the hidden variables in the data.

Surprisingly, the networks generated in this study show sufficient accuracy when compared to previous work and existing biological databases such as BIOGRIDE. Also, validation of the generated network using popular validation algorithms such as MCODE and NetworkAnalyzer adds more credibility to our algorithm. The data used in the validation step is not used for modelling. On the other hand, putative modules were recovered from our method, which suggests the need for more analysis to recover and test unknown complex modules.

We implemented the algorithm in Java. The program is open source and can be obtained from the authors.

## Competing interests

The authors declare that they have no competing interests.

## Authors' contributions

The initial idea of the algorithm was developed by all the authors. FA developed and tested the software. All the authors wrote and approved the manuscript.
